# Interpretation of Quantities Displayed in Pictorial Charts

**DOI:** 10.3389/fpsyg.2021.609027

**Published:** 2021-02-25

**Authors:** Tobias Rolfes

**Affiliations:** IPN – Leibniz Institute for Science and Mathematics Education, Kiel, Germany

**Keywords:** pictorial charts, illusion of linearity, problem-solving, cognitive processing, statistical literacy, data visualization

## Abstract

This brief research report presents an experiment investigating how people interpret quantities displayed in pictorial charts. Pictorial charts are a popular form of data visualization in media. They represent different quantities with differently scaled pictures. In the present study, 63 university students answered a 12-item questionnaire containing three different pictorial charts. The study aimed to evaluate how individuals perceive the quantities in the pictorial charts intuitively. Therefore, the students’ answers were not rated as correct or incorrect. Instead, it was analyzed which functional relationship between scale factor and estimated quantity best described people’s interpretation of pictorial charts. The experiment showed that, on average, a model assuming a quadratic relationship fitted best. This result deviates from research that found an overgeneralization of linearity when students compare the areas of two mathematically similar shapes. It may be that the routines for the interpretation of pictures differ considerably depending on whether a person must calculate a quantity arithmetically or is prompted to estimate the quantity based on visual perception.

## Introduction

Data and data analyses play an important role in decision making in modern society. Consequently, print media try to convey data of public relevance in graphically appealing and reader-friendly data visualizations. These graphics are becoming increasingly popular, graphically elaborate, and complex and are nowadays subsumed within the term *information graphics* or *infographics* (e.g., Cairo, 2013).

**FIGURE 1:**
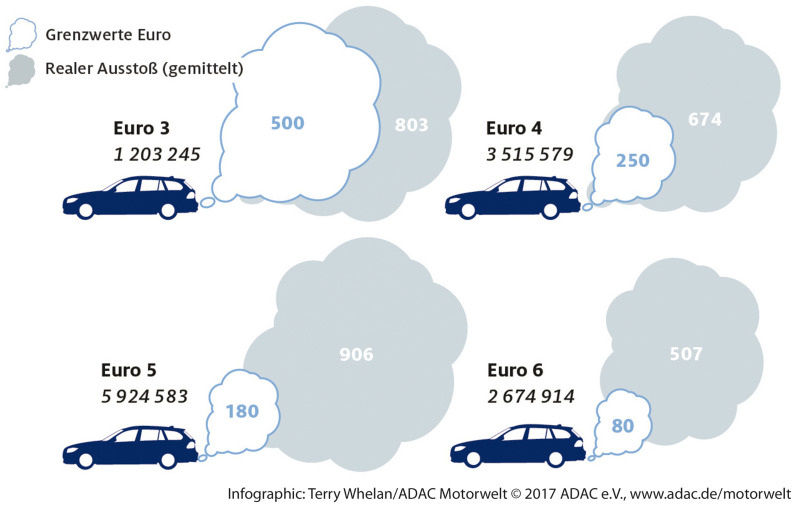
Pictorial chart of the nitrogen oxide emission of cars in a German magazine (Allgemeiner Deutscher Automobil-Club, 2017, p. 14). Copyright 2017 by Allgemeiner Deutscher Automobil-Club. Reprinted with permission (Grenzwert Euro: threshold value Euro norm, realer Ausstoβ: actual emission).

A specific and popular form of an infographic is a *pictorial chart* (Huff, 1954). It uses a picture related to the data to make the data presentation more aesthetically pleasing. Different quantities are displayed by scaling the picture up or down. Figure 1, which compares the nitrogen oxide emissions of different types of cars, provides an example of a pictorial chart. White clouds display the threshold values for nitrogen oxide emissions, and gray clouds represent the cars’ actual emission values. The “larger” the cloud, the greater the represented quantity of emissions.

**FIGURE 2:**
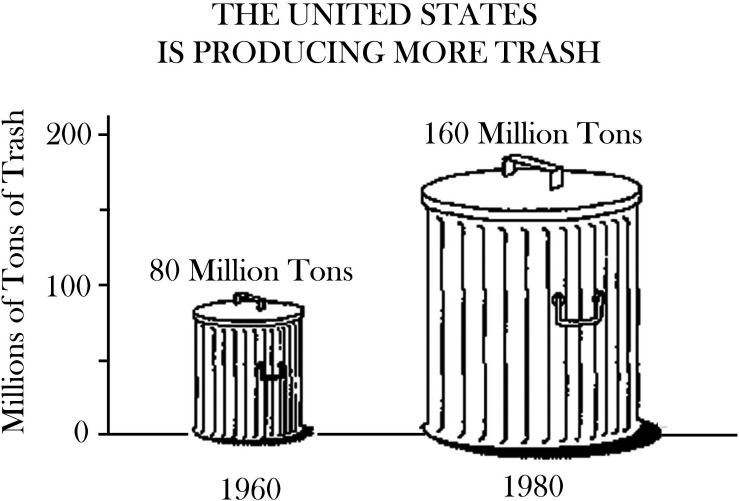
Stimulus for a standardized test item in a task: “The picture above is misleading. Explain why” (National Assessment of Educational Progress NAEP, 1992). Material of the National Assessment of Educational Progress is public domain.

An appealing graphical design for a chart can make data more accessible. However, graphical features can create a distorting visual impression and mislead the reader. Therefore, various countries’ curricula and standards in mathematics require students to have the ability to judge statistical data visualizations and to be able to identify misleading data displays (e.g., National Council of Teachers of Mathematics, 2000). Consequently, standardized testing includes the evaluation of pictorial charts (cf. Figure 2). To evaluate whether a pictorial chart is misleading, it is worth knowing how people interpret this form of data visualization. Do individuals base their interpretation on the real-life volume of the garbage can? Do they consider the covered area on the paper? Or do they only compare the height of the garbage cans?

First, this brief research report reflects from a theoretical perspective upon how people interpret pictorial charts and reviews empirical results from mathematics education and psychology that could substantiate different assumptions. Subsequently, the paper presents an empirical study investigating how students interpret pictorially displayed quantities in pictorial charts. Finally, in the “Discussion” section, the paper tries to explain the different research results by suggesting that whether people have to assess pictures analytically or perceptually might have a substantial effect. Furthermore, it is argued that given the increasing variety and importance of data visualization in public life, mathematics education should pay more attention to popular or novel forms of data visualizations.

## Theoretical Background

A pictorial chart is based on a similarity transformation. A graphic designer uniformly scales a picture A by a factor to generate a mathematically similar picture B. Tufte (2001) pointed out that in data visualization, the visual representation should be consistent with the numerical representation. For pictorial charts, Tufte’s claim raises the question of how people interpret visual representations and whether people’s interpretation is consistent with the intended numerical representation.

Therefore, one can try to generate assumptions about how people interpret pictorial charts by exemplarily analyzing an item (Figure 2) from a standardized test of the National Assessment of Educational Progress (NAEP). This item (National Assessment of Educational Progress NAEP, 1992) represents two quantities (the number of tons of trash produced in the United States in the years 1960 and 1980) using two perspective drawings of a garbage can. The students had to explain why the chart was misleading. The expected answer was based on mathematical considerations concerning the displayed objects’ volume in the real world. If the length, width, and height of a three-dimensional object are doubled, the volume increases eightfold. In other words, the relationship between the scale factor and the volume is cubic.

While the mathematics of volume is clear, this might not be the way people interpret the pictures of the garbage cans. If readers perceptually evaluate pictures based on the area covered by ink, the garbage can of 1980 extends over four times as much area as the garbage can of 1960. Generally speaking, the area of a shape quadruples if the shape’s length and width are doubled, due to a quadratic relationship between the scale factor and the area. There is also a third manner in which people could interpret the pictorial chart. In Figure 2, one could argue that the garbage cans are just replacing the bars of a bar chart to make the chart more appealing to the reader. Therefore, it might be possible that the reader, in the same way as when reading a bar chart, takes only the garbage cans’ height into account.

This ambiguity begs the question whether empirical research provides evidence of how readers evaluate pictorial charts. Empirical research that explicitly addressed readers’ interpretation of pictorial charts could not be found. However, research results from mathematics education and psychology may substantiate some theoretical assumptions outlined above.

### Results From Related Empirical Research

Since most pictorial charts rely on displaying quantities via representations of two- or three-dimensional objects, the reader’s ability and strategies to deal with measures (length, area, and volume) might influence the interpretation of pictorial charts. However, the ability to calculate these measures exactly does not play a significant role when interpreting pictorial charts. Instead, the ability to estimate measures might be crucial.

One strategy for estimating measures is the reference point strategy (Joram et al., 2005), that is, mentally comparing an object whose measurement is known with an object whose measure has to be estimated. When one estimates the length of a line by sight, for example, the empirical results turned out to be relatively clear-cut. Participants perceived lengths in a linear manner: that is, a line twice as long as another line was perceived to be twice as long (Stevens, 1975; Hartley, 1981). When a two-dimensional object was used and the size had to be estimated, the results were ambiguous. Stevens and Guirao (1963) used a square as the stimulus and found that doubling the side of the square resulted in a perceived *apparent size* 2.6 times as large. In two experiments Schneider and Bissett (1988) found that people estimated areas approximately correctly or slightly underestimated the area, whereas the participants consistently underestimated volumes. Based on his experiments, Morgan (2005) suggested that people apply various heuristics for estimating areas by combining width and height estimates. Investigating bubble charts, Raidvee et al. (2020) concluded that the human visual system does not perceive bubbles or discs in terms of their area but judges their size closer to their radius or diameter. These results indicate that people’s quantity estimation is not stable. Therefore, Joram et al. (1998) concluded that “measurement estimation is a highly volatile process, and easily influenced by the to-be-estimated objects” (p. 417).

A further research strand that relates to the interpretation of pictorial charts is research on problem-solving. In one experiment, De Bock et al. (1998) asked seventh-graders to solve word problems that required comparing areas. The students had to calculate how many hours it would take to fertilize a square piece of land with a side 600 m in length if a square piece of land with a side 200 m in length took 8 h to fertilize. The task was accompanied by scale drawings of the two square pieces of land. The results showed that most students assumed a linear relationship and answered 24 h; only 8% of them detected the quadratic relationship and solved the item correctly by answering 72 h. Solution rates for word problems that required an area comparison of two circles (5%) or two mathematically similar but irregular plane shapes (1%) were even lower than the rates for comparisons based on squares. In a replication study with tenth-graders, the solution rates were higher but still low (square: 39%, circle: 21%, irregular shape: 7%). The assumption of a linear relationship in situations that are based on nonlinear relationships has been termed the *illusion of linearity*. This phenomenon has been replicated in several studies (e.g., De Bock et al., 2002; Vlahović-Štetić et al., 2010) and could be demonstrated even among university students (Esteley et al., 2010).

### Present Study

In summary, the theoretical analyses and the empirical results in mathematics education and psychology show that it is still unclear how people interpret pictorial charts. That is, the question is the quantity *Q*_2_ that readers will assign to picture B when a picture A with a known quantity *Q*_1_ is uniformly scaled by a factor *s* and results in picture B. To evaluate whether a pictorial chart is misleading, one should know how people “read” a pictorial chart. Assuming that the processing of pictorial charts in media is based on intuitive heuristics that people quickly perform, the present study focuses on this *System 1* (Kahneman, 2011). The study did not assess whether the participants’ cognitive processes were correct or incorrect but aimed to describe the participants’ perception non-judgmentally. Therefore, the study investigated the functional relationship between the scale factor and the individually perceived quantity.

Some assumptions could be derived from the presented theoretical background. If readers apply an approximately linear relationship (as discovered in research about the overgeneralization of linearity) between the scaling factor and the quantity *Q*_2_, it results in the rule *Q*_2_ ≈ *s* ⋅ *Q*_1_. If people base their judgment on the perceptual aspects of interpreting a picture as a two-dimensional object (cf. summarized research above about the perception of areas), an approximately quadratic relationship could be assumed, that is, *Q*_2_ ≈ *s*^2^ ⋅ *Q*_1_. If a pictorial chart consists of perspective pictures of three-dimensional objects (e.g., photographs or perspective drawings of garbage cans), a spatial interpretation based on an approximately cubic relationship is possible (*Q*_2_ ≈ *s*^3^ ⋅ *Q*_1_). The NAEP coding guide, for example, evaluates this approach as the only correct solution for interpreting three-dimensional pictures. Although these three options would provide a clear-cut theoretical explanation for their occurrence, different exponents (e.g., 1.6 or 2.4) in the power functions could not be ruled out. Therefore, it appears reasonable to replace the exponent with a variable *b* so that the rule results in the general equation *Q*_2_ = *s^b^* ⋅ *Q*_1_.

The research questions for this study were: (RQ1) When one views a pictorial chart in which a quantity *Q*_2_ is displayed via scaling a picture representing the known quantity *Q*_1_ with a scaling factor *s*, which value *b* in the power function *Q*_2_ = *s^b^* ⋅ *Q*_1_ best describes a person’s interpretation of a quantity *Q*_2_? (RQ2) Does the value *b* vary substantially between persons? (RQ3) Does the value *b* depend on the picture? (RQ4) Does the value *b* depend on whether the pictures were enlarged or reduced?

## Materials and Methods

### Participants

This study drew the participation of 63 mathematics teacher-education students from a German university (primary and secondary school education) with an average age of *M* = 21.5 (*SD* = 2.0). Since most of the students aspired to the primary school teaching profession, female students predominated in the sample (58 females, 5 males). The students had not received instruction on the study’s topic, nor was the topic explicitly taught in any course. The students were recruited during ordinary course time and did not receive a financial incentive. Ethics approval was obtained from the students.

### Materials

**FIGURE 3:**
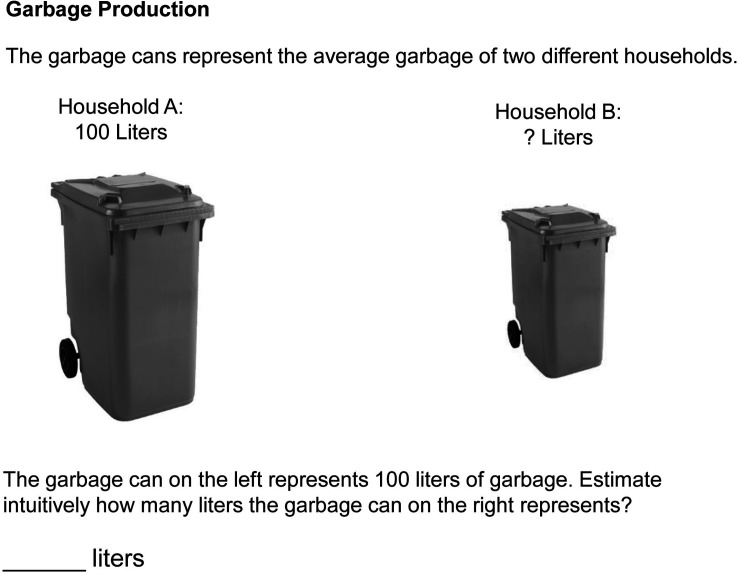
Item of the Garbage test translated into English: The garbage can on the left is scaled by the factor 0.7.

The questionnaire consisted of 12 items (three testlets with four items each). A specific picture formed the basis of every testlet. The three pictures differed in their level of realism and the extent to which they can be interpreted two- or three-dimensionally. In the *CO*_2_ testlet, a line drawing of a cloud represented the amount of carbon dioxide emitted by a factory. This line drawing could be interpreted two-dimensionally as the cross-section of a cloud. A three-dimensional interpretation was also possible by taking into consideration the real-life nature of a cloud and the overlapping lines. However, how deep the cloud is perceived depends on the reader’s interpretation. In the *Garbage* testlet (Figure 3), a photograph of a garbage can depicted the amount of garbage produced by a household. The photograph was used to stimulate a three-dimensional interpretation. In the *Sugar* testlet, a perspective line drawing of a sugar lump in cavalier projection displayed the amount of sugar a person consumes. Like the Garbage testlet, the Sugar testlet should stimulate a three-dimensional interpretation. A picture of a cloud, a garbage can, or a sugar lump on the left-hand side displayed a base quantity of 100 units in each testlet. On the right-hand side, the same picture was uniformly scaled by a specific factor (e.g., 0.7 or 1.6, with the complete item booklet provided in Supplementary Material, File 2, Chapter A). Different scale factors were used to derive general rules from the data about the relationship between scale factors and perceived quantities. The pictorial charts did not contain an ordinate, and the pictures were not placed on a horizontal baseline. The participants had to intuitively estimate the quantity represented by the picture on the right because the aim of the experiment was to determine the readers’ innate interpretation of the pictorial chart. Each testlet comprised four comparisons. The 12 items were presented in a fixed order (CO_2_, Garbage, Sugar). The scale factor *s* in the experiment ranged from 0.3 to 1.9.

### Administration

The students answered the questionnaire with paper and pencil. They were told that the questions dealt with intuitive estimation. Hence, the students were asked to refrain from using a calculator or a ruler and from performing calculations manually. It took the students between 5 and 10 min to complete the questionnaire.

### Data Analysis

Each of the three testlets contained four items, resulting in 12 items per person. The data analysis aimed to determine the functional relationship between scale factor and a person’s estimate. It was assumed that the estimation follows a power function *Q*_2_ = *s^b^* ⋅ *Q*_1_ (cf., section “Theoretical Background”). In the experiment, *Q*_1_ always equaled 100, so the equation becomes *Q*_2_ = 100 *s^b^*. Taking logarithms of the equation, we get: $\text{log}\,\left(\frac{Q_{2}}{100}\right)$ = *b* ⋅ log (*s*). Therefore, to determine the exponent *b*, we apply a linear regression *y* = *bx* + *e* with the logarithm of the scale factor, log(s), as the independent variable *x* and the logarithm of *Q*_2_ divided by 100, $\text{log}\,\left(\frac{Q_{2}}{100}\right)$, as the dependent variable *y*, and *e* as the residual. In the equation *y* = *bx* + *e*, the value of the regression slope *b* equals the exponent *b* in the power function. The regression intercept is zero.

The data had a multilevel structure because the responses were nested within testlets and persons. Classical regression analysis cannot account for the dependence on the responses within persons. Therefore, multilevel models (cf. Gelman and Hill, 2006) were applied to all analyses.

First, a two-level-approach (responses nested within persons) was applied. To answer the first research question, an average value for the exponent *b* for all participants across all items was estimated (Model 1: fixed slope). To evaluate whether the exponent *b* varies among individuals (research question 2), a value for the exponent *b* for every participant across all 12 items was determined (Model 2: random slope). In Model 3, the testlet structure was taken into account (answers nested within testlets and individuals). That is, this model estimated whether the testlet (CO_2_, Garbage, and Sugar) had an effect on the exponent *b* (research question 3). In Model 4, a further fixed effect (enlargement or reduction) was added to Model 3 to evaluate RQ4. A detailed description of the multilevel analyses can be found in Supplementary Material, File 2, Chapter B.

Outliers were replaced with missing values before conducting the multilevel analyses. A value was defined as an outlier if picture B was scaled down, but a student estimated a value bigger than 100 and vice versa.

## Results

Every participant answered all 12 items, resulting in 756 item responses (cf. descriptive statistics in Table 1). Six item responses were identified as outliers and replaced with missing values.

**TABLE 1:** Descriptive information concerning the items.

				Estimated quantity for Q_2_						
Item No.	Test	Task	Scaling factor	If *b* = 1 (linear)	If *b* = 2 (quadratic)	If *b* = 3 (cubic)	*N*	*M*	*SD*	95% CI
1	CO_2_	a	0.9	90	81	73	63	80.9	7.4	(79.0, 82.7)
2		b	1.3	130	169	220	62	177.5	59.6	(162.4, 192.7)
3		c	0.3	30	9	3	60	10.9	4.1	(9.8, 11.9)
4		d	1.6	160	256	410	63	290.2	98.3	(265.4, 314.9)
5	Garbage	a	0.7	70	49	34	63	54.5	16.1	(50.4, 58.6)
6		b	1.5	150	225	338	62	189.3	47.0	(177.3, 201.2)
7		c	1.9	190	361	686	63	344.4	147.4	(307.2, 381.5)
8		d	0.5	50	25	13	62	28.4	12.0	(25.3, 31.4)
9	Sugar	a	1.4	140	196	274	63	195.0	52.1	(181.9, 208.1)
10		b	0.8	80	64	51	63	67.9	13.2	(64.6, 71.2)
11		c	0.4	40	16	6	63	19.2	9.0	(16.9, 21.5)
12		d	1.8	180	324	583	63	365.6	180.9	(320.1, 411.2)

*The six outliers were eliminated before calculating the descriptive statistics.*

Regarding the first research question, the aim was to identify in the power function *Q*_2_ = 100*s^b^* the exponent *b* that best described the participants’ perception of the quantities in the pictorial charts. Using logarithms of the estimation values and the scale factors caused the slopes in the multilevel analyses to equal the searched value *b* (cf. “Materials and Methods” section). The first multilevel model (Model 1) with fixed slopes (i.e., assuming that the value *b* did not vary among participants) resulted in a value 1.92 for the exponent *b*, 95% CI (1.87, 1.96). The explained variance in this model was 91% (Pseudo-*R*^2^). That is, on average, the interpretation of the displayed quantities in a pictorial chart followed approximately a quadratic relationship.

The second research question dealt with the question whether the value *b* varied among participants. The second multilevel model (Model 2) with random slopes showed that the exponent *b* varied significantly among the participants, as a model comparison between the first and second model showed. The values ranged from the lowest value 1.4 to the highest value 2.7. Thirty-two of the participants (i.e., 51%) had an exponent between 1.75 and 2.25, that is, an approximately quadratic relationship. Several participants showed values in between two whole numbers for the exponent *b*. Twenty-three participants (37%) had an exponent between 1.25 and 1.75, and 8 participants (13%) had exponents between 2.25 and 2.75.

RQ3 addressed the issue of whether the estimation process depended on the picture in a pictorial chart. Therefore, in a three-level model (Model 3), a testlet effect was estimated by assuming a fixed effect of the testlet. This model fitted significantly better than Model 2, χ^2^(1) = 100.1, *p* < 0.001, and the explained variance was enhanced from 93.5 to 95.6%. Although the testlet effect was significant, its size was rather small. This model’s average value for the exponent *b* also was 1.92, 95% CI (1.83, 2.01). For the CO_2_ testlet, the value 0.04, 95% CI (0.01, 0.07) has to be added to this exponent; for the Garbage testlet, the value 0.06, 95% CI (–0.10, –0.03), has to be subtracted, and for the Sugar testlet, the value 0.02, 95% CI (–0.01, 0.05), has to be added.

Finally, the question was whether the estimation process was influenced by whether the pictures were enlarged or reduced (RQ 4). Model 4 did not improve the model fit in comparison with Model 3, χ^2^(1) = 0.13, *p* = 0.72, and the fixed effect (enlargement or reduction) did not significantly differ from zero, 95% CI (–0.09, 0.06). Detailed information about the results of the multilevel analyses can be found in Supplementary Material, File 2, Chapter B.

## Discussion

The study showed that the participants applied, on average, an approximately quadratic relationship (*b* = 1.92) between the scale factor and the estimated quantity. That is, generally, the participants estimated the quantity in a pictorial graph based on the area of the picture. The *b*-values, however, differed among participants. None of the 63 participants could be identified to operate with a linear relationship or a cubic relationship when dealing with pictorial charts. The majority based their judgment on an approximately quadratic relationship (51%). A considerable proportion of the students (37%) had an estimation process in between a linear and a quadratic relationship. These students might have intended to estimate the area but did it in a biased manner because research has shown many people underestimate areas (e.g., Stevens, 1975; Raidvee et al., 2020). That means that the interpretation of pictorial charts is probably based on the visual perception of the ink-covered area rather than the result of an analytical process. As for the research question on whether the picture influences the estimation process, the experiment showed that the exponent *b* varied among testlets, although the difference was relatively small (between 1.84 and 1.95). Therefore, the type of picture (i.e., a perspective or non-perspective line drawing or a photograph) did not substantially influence the estimation process, nor did it have an effect on whether the picture was an enlargement or a reduction.

Within the context of the theoretical considerations presented and prior empirical results from mathematics education and psychology, the experiment revealed some surprising results. First, the assumption that, in general, participants apply spatial considerations to a two-dimensional picture of a three-dimensional object cannot be corroborated. Second, the present study’s results seem to deviate from the results of several experiments that found a robust overgeneralization of linear models when pictures were provided in problem-solving tasks. However, the study’s empirical results align most closely with the findings regarding the perception and estimation of areas.

These results raise the question whether the different results from the research on the illusion of linearity and the present experiment can be reconciled. In research concerning the overgeneralization of linear models, the students were asked to *calculate* the area or an indirect measure of the area (e.g., the time to fertilize a piece of land). The students had to use arithmetic operations (e.g., addition and multiplication) to solve the problem. Research has shown that students looked for analogies when asked to solve a new problem (Gentner et al., 2001). Proportional reasoning is often successful in mathematics. Students seem to rely on this heuristic in mathematics even when a closer look at the picture of the square of land could reveal its incorrectness. In the present study, however, students were requested to *estimate* the quantity of the pictures displayed based on their perception. The participants were explicitly asked to refrain from calculations.

The assumption that it matters whether students have to *estimate* the quantity represented based on visual perception or to *calculate* the quantities using arithmetic operations can be supported by theories concerning information processing of texts and pictures (e.g., Mayer, 2014). They assume that cognitive processing differs according to whether it occurs on the symbolic (e.g., words, texts, mathematical signs) or the pictorial channel. In research detecting the illusion of linearity, students were urged to work on the symbolic channel because symbolic information was given (e.g., the length of the side of a square of land), and calculations were required. In the present experiment, however, the students were not provided with numerical information about the figure’s length or width. They were nudged to process the pictures on the pictorial channel and to assign quantities based on their visual perception. Therefore, it seems reasonable to assume that the processing channel has a decisive effect on the results.

With respect to the learning and teaching of mathematics, the experiment showed that tasks such as those in Figure 2 are problematic when the aspect of “misleading” is only judged theoretically as the NAEP coding guide does. The present experiment showed that even mathematically inclined persons did not base their quantity interpretations on real-world volume. Therefore, students must be sensitized to this issue on a more sophisticated level. However, further research in this field is necessary to infer more specific knowledge that can be taught in school about the interpretation of novel forms of data visualizations.

However, some limitations should be mentioned. First, the sample with mathematics teacher education students is selective. The question is whether the findings are generalizable to different samples (e.g., younger students or less mathematically educated individuals). It could be possible that mathematically inclined individuals interpret pictorial charts differently from people without a mathematics background. Furthermore, there could have been a priming effect, as the clouds were always presented first and could have prompted an estimate based on areas. Moreover, in future experiments, the picture’s effects in pictorial charts should be investigated further by using diverse types of pictures and varying them systematically. In a subsequent study, the presented results should be replicated using a more diverse sample and different pictures. A possible sequencing effect should be controlled for by permuting the testlets. Furthermore, it would be interesting to investigate whether providing a legend in a pictorial chart would affect the reader’s interpretation.

## Conclusion

Some conclusions can be drawn from the present study in terms of the design of pictorial charts. Readers do not seem to interpret two-dimensional pictures of three-dimensional objects spatially. Therefore, chart designers probably should refrain from using pictures of three-dimensional objects to display quantities. With regard to the growing popularity of infographics (e.g., Yau, 2011; Cairo, 2013) and software for data visualizations (e.g., GapMinder), the investigation of how individuals perceive these visualizations is also an important educational aspect. Therefore, mathematics education should also integrate teaching and research on nonstandard, novel forms of data visualizations because they are becoming increasingly prevalent in everybody’s life.

## Data Availability Statement

The present study’s original data are included in the Supplementary Material, File 1, further inquiries can be directed to the author.

## Ethics Statement

Ethical review and approval was not required for the study on human participants in accordance with the local legislation and institutional requirements. The participants provided their written informed consent to participate in this study.

## Author Contributions

The author confirms being the sole contributor of this work and has approved it for publication.

## Conflict of Interest

The author declares that the research was conducted in the absence of any commercial or financial relationships that could be construed as a potential conflict of interest.
