# Supplementary material for: Interpretation of Quantities Displayed in Pictorial Charts
Source: Front Psychol. 2021 Feb 25;12:609027. doi: 10.3389/fpsyg.2021.609027 (PMC7959779; doi:10.3389/fpsyg.2021.609027)
Supplement: Supplementary file 2 [file Data_Sheet_2.PDF]

Supplementary Material, Chapter A: Items (translated into Englisch)

Carbon Dioxide Emission

The clouds represent the carbon dioxide emission of two different factories.

a)

Factory A:
100 Tons of
Carbon Dioxide

Factory B:
? Tons of
Carbon Dioxide

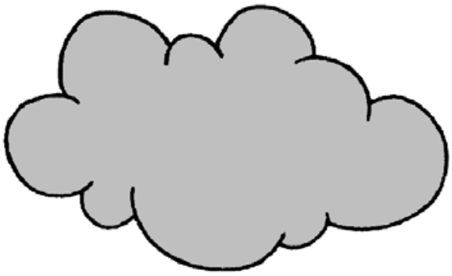

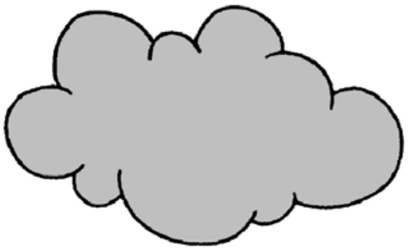

The cloud on the left represents 100 tons of Carbon Dioxide. Estimate intuitively how many tons of carbon dioxide the cloud on the right represents.

______ ton of carbon dioxide

b)

Factory A:
100 Tons of
Carbon Dioxide

Factory C:
? Tons of
Carbon Dioxide

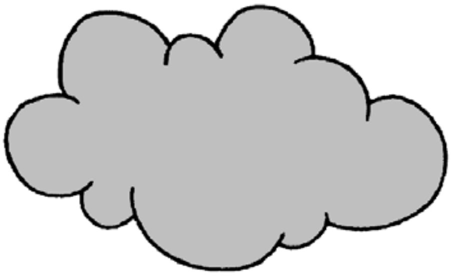

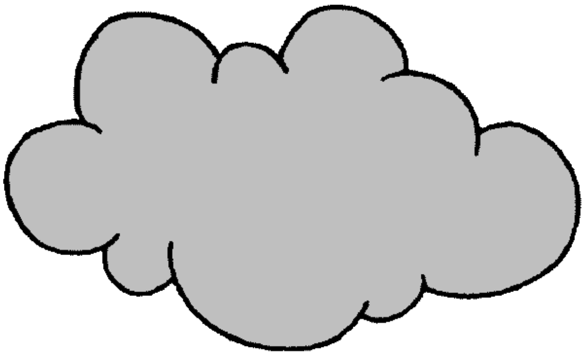

The cloud on the left represents 100 tons of Carbon Dioxide. Estimate intuitively how many tons of carbon dioxide the cloud on the right represents.

______ ton of carbon dioxide

c)

Factory A:
100 Tons of
Carbon Dioxide

Factory D:
? Tons of
Carbon Dioxide

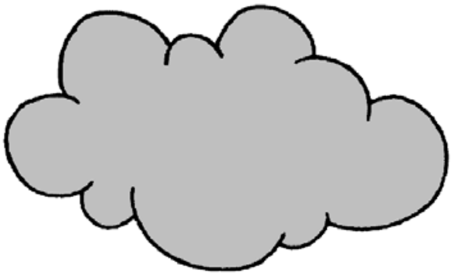

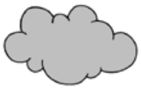

The cloud on the left represents 100 tons of Carbon Dioxide. Estimate intuitively how many tons of carbon dioxide the cloud on the right represents.

______ ton of carbon dioxide

d)

Factory A:
100 Tons of
Carbon Dioxide

Factory E:
? Tons of
Carbon Dioxide

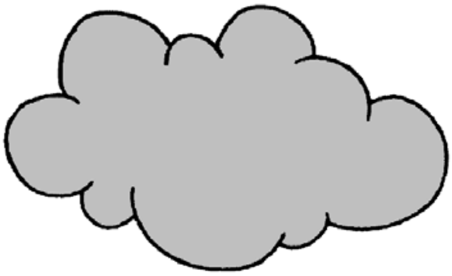

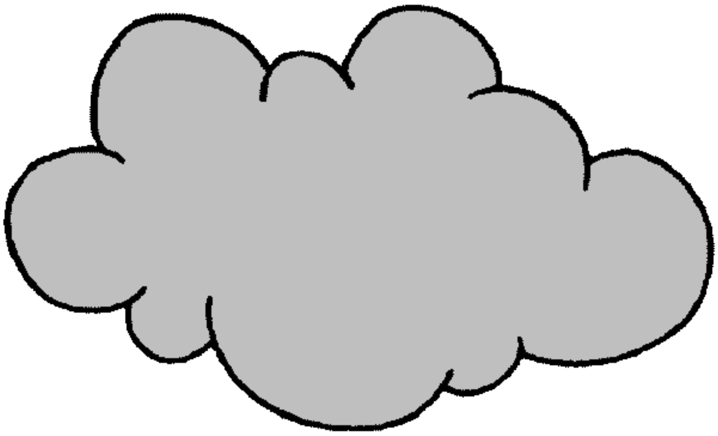

The cloud on the left represents 100 tons of Carbon Dioxide. Estimate intuitively how many tons of carbon dioxide the cloud on the right represents.

______ ton of carbon dioxide

Garbage Production

The garbage cans represent the average garbage of two different households.

a)

Household A:
100 Liters

Household B:
? Liters

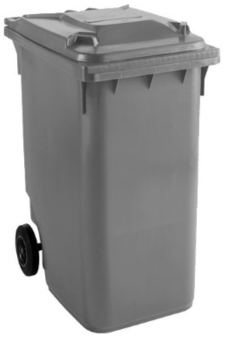

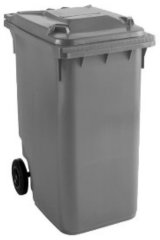

The garbage can on the left represents 100 liters of garbage. Estimate intuitively how many liters the garbage can on the right represents.

______ liters

b)

Household A:
100 Liters

Household C:
? Liters

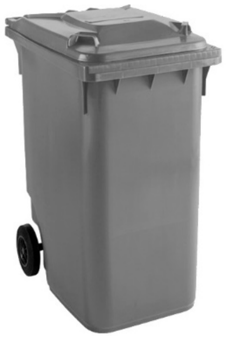

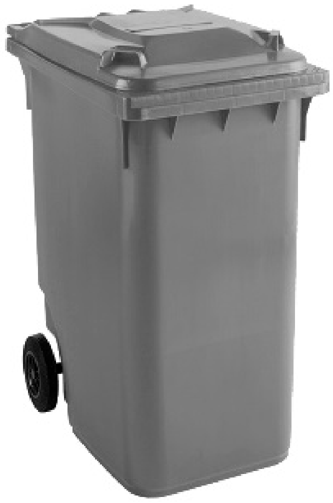

The garbage can on the left represents 100 liters of garbage. Estimate intuitively how many liters the garbage can on the right represents.

______ liters

c)

Household A:
100 Liters

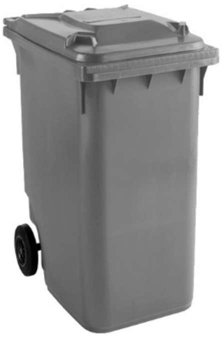

Household D:
? Liters

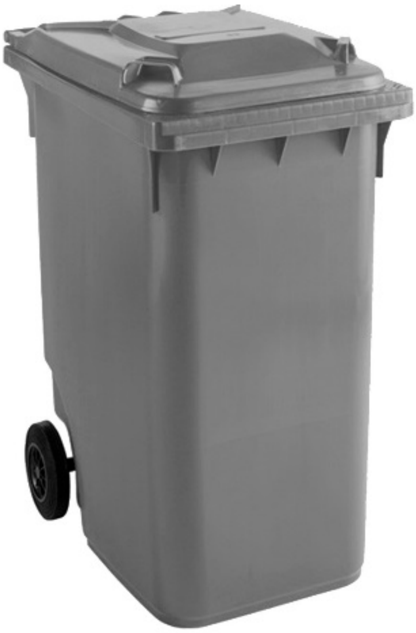

The garbage can on the left represents 100 liters of garbage. Estimate intuitively how many liters the garbage can on the right represents.

______ liters

d)

Household A:
100 Liters

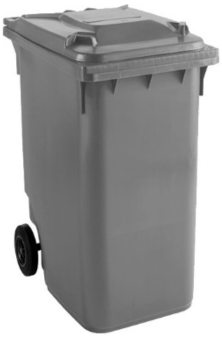

Household E:
? Liters

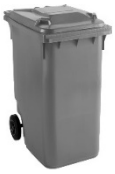

The garbage can on the left represents 100 liters of garbage. Estimate intuitively how many liters the garbage can on the right represents.

______ liters

Sugar Consumption

The objects represent the average daily sugar consumption per person in two different countries.

a)

Country A:
100 Grams

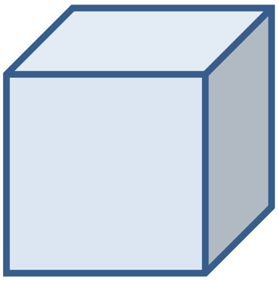

Country B:
? Grams

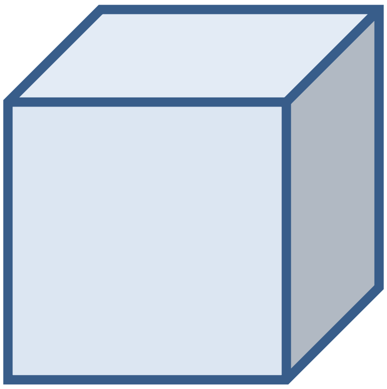

The sugar lump on the left represents 100 grams of sugar. Estimate intuitively how many grams of sugar the object on the right represents.

_______ grams

b)

Country A:
100 Grams

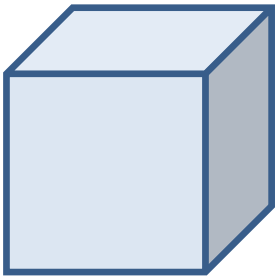

Country C:
? Grams

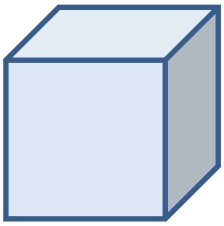

The sugar lump on the left represents 100 grams of sugar. Estimate intuitively how many grams of sugar the object on the right represents.

_______ grams

c)

Country A:
100 Grams

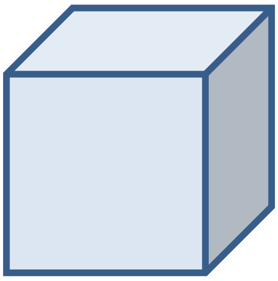

Country D:
? Grams

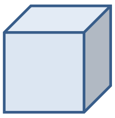

The sugar lump on the left represents 100 grams of sugar. Estimate intuitively how many grams of sugar the object on the right represents.

_______ grams

d)

Country A:
100 Grams

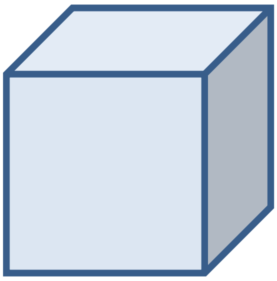

Country E:
? Grams

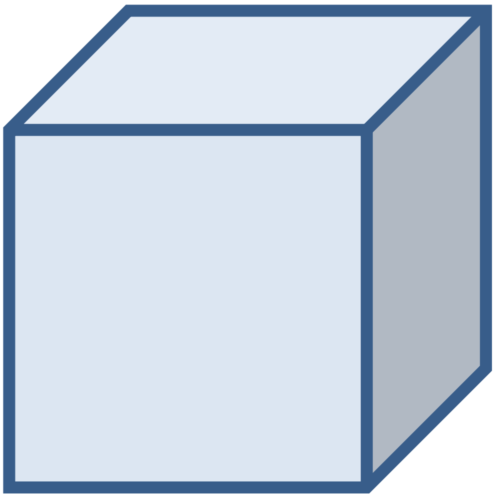

The sugar lump on the left represents 100 grams of sugar. Estimate intuitively how many grams of sugar the object on the right represents.

_______ grams

Supplementary Material, Chapter B: Multilevel analyses

First, models using a two-level-approach (responses nested within persons, cf. Fig. 1) were calculated. The null model $y_{ip} = e_{ip}$ (y_{ip}: the logarithm of the estimate by person *p* of item *i*; e_{ip}: residual) served as the baseline model and assumed no relationship between the scaling factor and a person's estimate. Introducing a fixed slope b into the null model's multilevel equation resulted in Model 1: $y_{ip} = bx_i + e_{ip}$ (x_i: logarithm of the scale factor of item *i*). This model assumed that the picture's scaling factor influences the student's estimate. Model 1, however, did not allow the slope b to vary among persons; that is, in the equation $Q_2 = 100s^b$, Model 1 assumed no differences in the exponent b among the students.

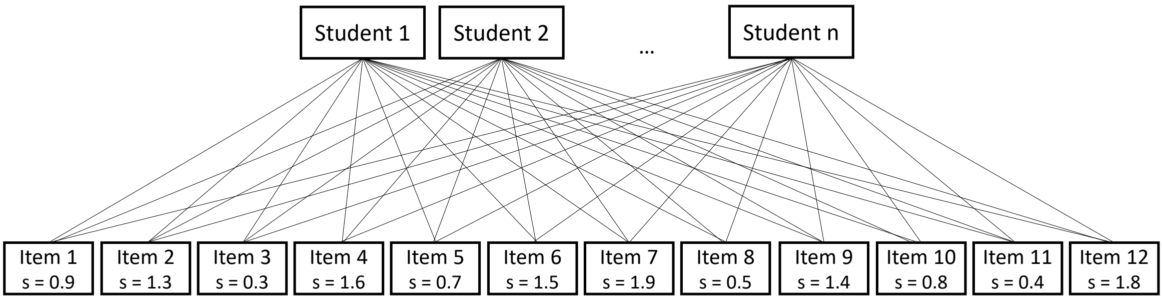

Figure 1. Two-level model.

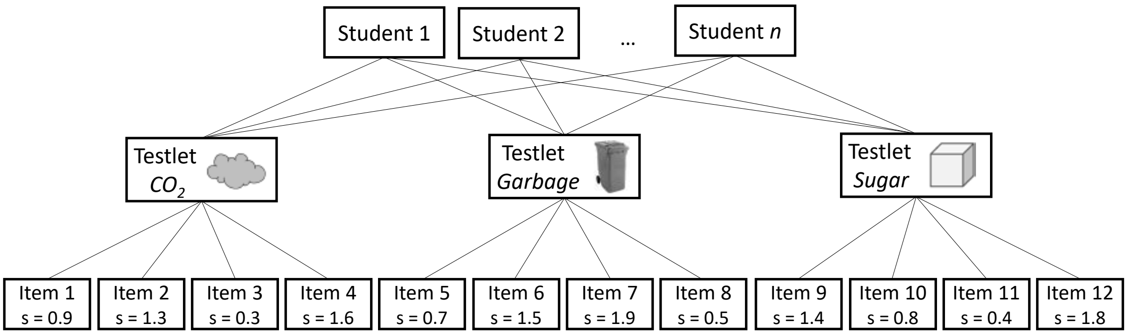

Figure 2. Three-level model.

The intercept was omitted in Model 1 because, as shown in the Method section, taking logarithms of $Q_2 = 100s^b$ lead to a linear regression with a zero intercept. In Model 2: $y_{ip} = (b + u_p)x_i + e_{ip}$, a random slope u_p was inserted; that is, the model considered students using different exponents when estimating Q_2 (cf. Fig. 3).

In Model 3, a three-level model (responses nested within testlets and persons, cf. Fig. 2) was specified, as three different pictures were used in the experiment. The different pictures could account for the variance in the responses: $y_{ip} = (b + v_t + u_p + w_{tp})x_i + e_{ip}$ (v_t: fixed effect of the three testlets; w_{tp}: random effect of persons for a testlet).

Finally, Model 4 extended Model 3 with a fixed effect of the dichotomous variable whether the picture in the pictorial chart was enlarged or reduced: $y_{ip} = (b + l + v_t + u_p + w_{tp})x_i + e_{ip}$ (l: fixed effect of the dichotomous variable enlargement, *yes* or *no*).

Table 1. Comparison of different multilevel models.

	Null Model	Model 1	Model 2	Model 3	Model 4
Fixed effect					
Exponent (b)	-	1.918	1.918	1.919	1.929
$\sigma(b)$	-	0.022	0.044	0.046	0.055
Testlet CO_2	-	-	-	0.040	0.048
σ(Testlet CO_2)	-	-	-	0.017	0.029
Testlet Garbage	-	-	-	–0.064	–0.058
σ(Testlet Garbage)	-	-	-	0.016	0.025
Testlet Sugar	-	-	-	0.023	0.030
σ(Testlet Sugar)	-	-	-	0.016	0.027
Enlargement (*yes*, *no*)	-	-	-	-	–0.014
σ(Enlargement)	-	-	-	-	0.040
Random effects					
Person: σ^2	-	-	0.102	0.083	0.083
Testlet: σ^2	-	-	-	0.099	0.099
Residual: σ^2	1.426	0.128	0.092	0.065	0.065
Number of observations	750	750	750	750	750
Number of groups: Testlet:Person	-	-	-	189	189
Number of groups: Person	63	63	63	63	63
Pseudo-R^2	-	0.910	0.935	0.954	0.954
Number of parameters	2	3	3	7	8
LogLik	-1197	-293	-224	-174	-174
Deviance	2395	585	449	348	348
AIC	2399	591	455	362	364
BIC	2408	605	468	395	401

Note. Model parameters were calculated using restricted maximum likelihood estimations.

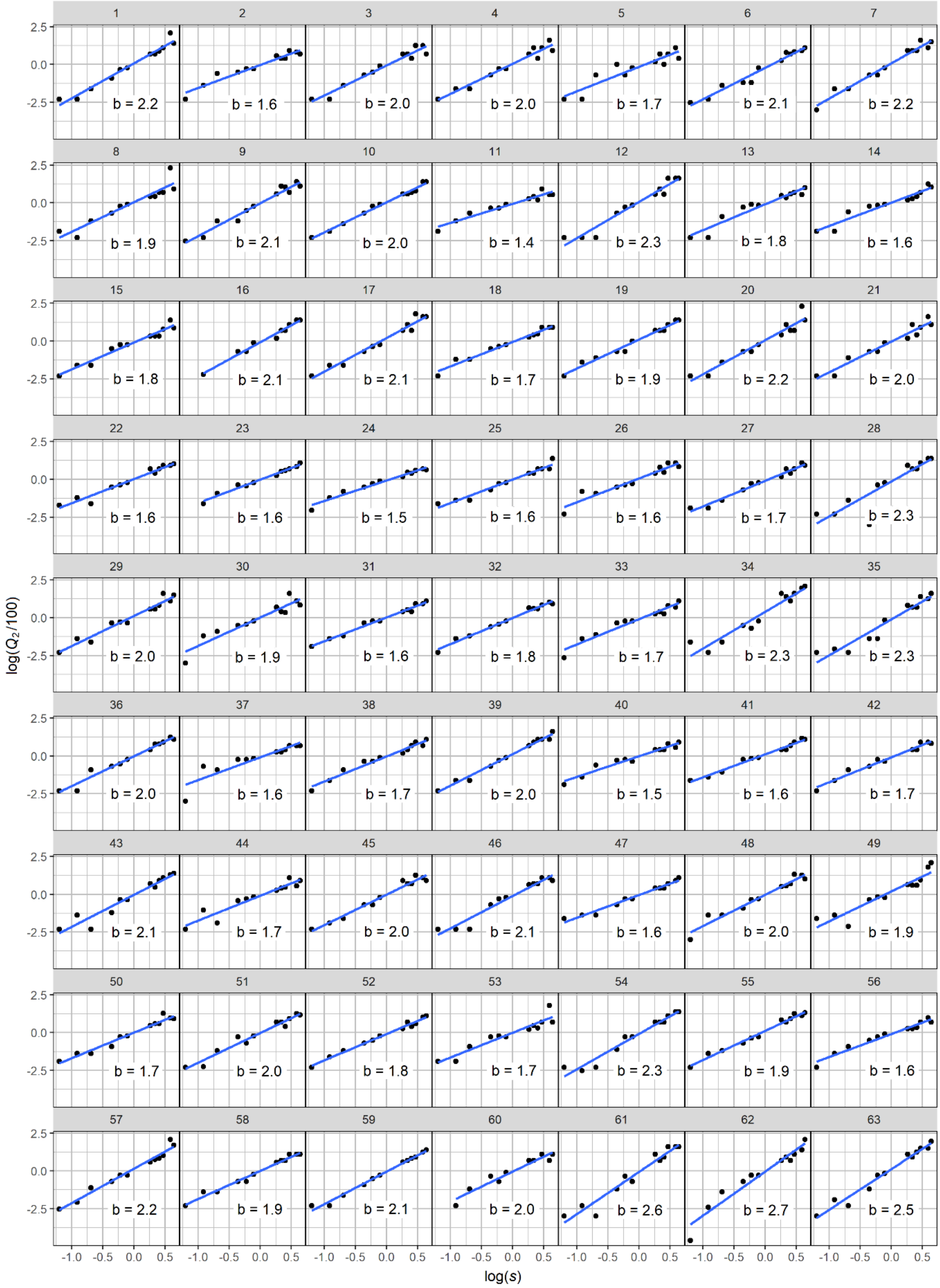

Figure 3. Scatterplots of the logarithm of the scale factor, log(s), and the logarithm of the student's estimates, log(Q_2/100), and the students' individual slopes b of Model 2.
